# Correlation between imaging-detected and pathological extranodal extension in a randomised trial in Human Papillomavirus-positive oropharyngeal cancer

**DOI:** 10.1038/s41416-025-03291-z

**Published:** 2025-11-27

**Authors:** Mererid Evans, Chris Hurt, Rhian Rhys, Abhishek Mahajan, Andrew McQueen, Joanna Dixon, Max Robinson, Neil Robinson, Keith Hunter, Adam Christian, Adam Jones, Aline Queiroz, Shao Hui Huang, Brian O’Sullivan, Joanna Canham, Christie Heiberg, Terry Jones

**Affiliations:** 1 Division of Cancer and Genetics, Cardiff University and Dept. of Clinical Oncology, Velindre University NHS Trust, Cardiff, UK; 2 Southampton Clinical Trials Unit, University of Southampton, Southampton, UK; 3 Deptartment of Radiology, Cwm Taf Morgannwg University Health Board, Abercynon, UK; 4 Imaging Department, The Clatterbridge Cancer Centre NHS Liverpool and Faculty of Health and Life Sciences, University of Liverpool, Liverpool, UK; 5 Department of Radiology, Newcastle Upon Tyne Hospitals NHS Foundation Trust, Newcastle, UK; 6 Department of Cellular Pathology, Newcastle Upon Tyne Hospitals NHS Foundation Trust, Newcastle, UK; 7 Liverpool Head and Neck Centre, Faculty of Health and Life Science, University of Liverpool, Liverpool, UK; 8 Department of Pathology, Cardiff and Vale University Health Board, Cardiff, UK; 9 Department of Oral Pathology, University of Sao Paulo, Sao Paulo, Brazil; 10 Department of Radiation Oncology, Princess Margaret Cancer Center, University of Toronto, Toronto, Canada; 11 Centre for Trials Research, School of Medicine, Cardiff University, Cardiff, UK

**Keywords:** Head and neck cancer, Surgical oncology, Cancer imaging

## Abstract

**Background:**

Imaging-detected and pathological extranodal extension (iENE, pENE) negatively impact prognosis in Human Papillomavirus (HPV)-positive oropharyngeal cancer (OPSCC), as reflected in future TNM staging updates. Correlation between iENE and pENE in HPV-positive OPSCC is currently unknown yet is vital to determine how iENE should be used to influence treatment decisions.

**Methods:**

PATHOS is a trial of de-intensified adjuvant treatment after transoral surgery for HPV-positive OPSCC. 291 consecutively recruited patients undergoing surgery at three UK centres were included. Pre-operative cross-sectional imaging (CT and/or MRI) was independently scored for iENE by 2 expert radiologists; pENE was scored by 2 expert pathologists.

**Results:**

Inter-rater agreement for iENE was fair in round 1 (Gwet’s AC: 0.34 (95%CI:0.26–0.41)) but improved to very good after second review (Gwet’s AC: 0.88 (95%CI:0.85–0.93), Agreement: 0.91 (95%CI:0.87–0.94)). Sensitivity of iENE for predicting pENE was relatively low (at best: 56.4% (95%CI:42.3–69.7) and specificity was high (at worst: 70.9% (95%CI:65.0–76.3)). Excluding cases with suboptimal image quality and recent core biopsy produced modest improvements in sensitivity (up to 59.4% (95%CI:40.6–76.3)) and specificity (up to 87.8% (95%CI:80.4–93.2)).

**Discussion:**

The high specificity could help select iENE-negative patients for surgery, but higher sensitivity is required before excluding surgery based solely on iENE positivity.

## Introduction

Human Papillomavirus (HPV)-positive oropharyngeal squamous cell carcinoma (HPV-positive OPSCC), predominantly affecting the tonsils and base of tongue, has gained significant attention over the last two decades because of its increasing incidence in developed countries, its younger demographic and its better treatment response and prognosis compared to smoking-related HPV-negative OPSCC [1]. To reflect its unique biology and prognosis, the 8^th^ edition American Joint Committee on Cancer (AJCC)/ Union for International Cancer Control (UICC) TNM staging manual published in 2017 introduced a new, distinct staging system for the clinical (cTNM) and pathological staging (pTNM) of HPV-positive OPSCC, which have been widely implemented [2]. There are proposals to further refine the staging of HPV-positive OPSCC in future to account for the influence of extranodal extension (ENE) on the outcomes of patients with HPV-positive OPSCC [3, 4].

Extranodal extension (ENE) refers to the growth of a nodal cancer metastasis beyond the confines of the capsule of a lymph node into surrounding tissues. It is a strong prognostic factor for non-HPV related head and neck squamous cell carcinoma (HNSCC), predicting for both regional recurrence and distant metastasis and is an indication for adjuvant chemoradiotherapy [5–7]. Clinically overt ENE (defined by physical examination and supported by radiological evidence) is included in the 8th edition clinical N (cN) classification of HPV-negative OPSCC, and pathological ENE (pENE), visible by the pathologist on microscopic examination of a resected cancer, is included in the 8th edition pathological N (pN) classification of all HNSCC, except HPV-positive OPSCC and nasopharyngeal carcinoma.

Over recent years, emerging data have suggested that ENE is a prognostic factor for HPV-positive OPSCC, as it is for non-HPV related HNSCCs. This appears to be true for ENE that is detected on imaging (imaging-detected ENE, or iENE) [8, 9] and ENE that is visible on pathological examination (pathological ENE, or pENE) [10–12]. The correlation between iENE seen on anatomic imaging, and pENE seen on pathological examination of a resected specimen is unknown and will not be determined for the majority of patients who undergo primary non-surgical treatment (radiotherapy or chemo-radiotherapy) for HPV-positive OPSCC. The correlation is vital to determine how iENE should be used to influence up-front multi-disciplinary treatment decisions; in particular, it is important to know whether iENE is an accurate enough predictor of pENE to justify signposting patients with iENE towards primary radiotherapy/chemo-radiotherapy, instead of surgery and adjuvant radiotherapy/chemo-radiotherapy. The relationship between iENE and pENE can only be determined in a surgically treated cohort of patients.

PATHOS is a phase III randomised controlled trial (RCT) of risk-stratified, reduced intensity adjuvant treatment in patients with HPV-positive OPSCC, who have undergone transoral surgical resection of the primary tumour, and a neck dissection [13]. The trial, which completed recruitment on 31 October 2024, provides a perfect vehicle for testing the correlation between iENE and pENE in a well-annotated, prospective cohort of patients with HPV-positive OPSCC, all of whom have undergone baseline imaging prior to surgery, followed by histological examination of the resected surgical specimen.

## Patients and methods

### Study population

PATHOS (ClinicalTrials.gov: NCT02215265, Supplementary Fig. S1) is a multicentre, open-label, parallel-group, phase III RCT for patients with transorally resectable T1-3 N0-N1 (TNMV8) HPV-positive OPSCC. A total of 1349 participants have been recruited from the UK, US, France, Germany and Australia and follow-up for the primary survival endpoint is ongoing. Recruited participants undergo transoral primary tumour resection using either a robot or laser and neck dissection, followed by post-operative risk group allocation and randomisation.

291 patients who were consecutively recruited into PATHOS and had surgery between 02/11/2015 and 26/02/2024 at three regional UK centres (Liverpool University Hospitals NHS Foundation Trust, Newcastle upon Tyne Hospitals NHS Foundation Trust, South-East Wales [comprising Cwm Taf Morgannwg, Cardiff and Vale and Aneurin Bevan University Health Boards]), with available imaging and pathologic specimens for iENE and pENE assessment respectively, were included in this PATHOS-ENE sub-study. Patients who had lymph node removal prior to imaging were excluded.

### Imaging-detected Extranodal Extension (iENE) assessment

Contrast-enhanced, cross-sectional imaging (CT and/or MRI neck) was performed pre-operatively according to local protocols in each participating hospital. Four specialist head and neck radiologists (blind to pENE assessment) undertook iENE reviews – 1 from Liverpool and 1 from South-East Wales (team 1) and 2 from Newcastle (team 2). Each case was independently reviewed for iENE by 2 radiologists and scored for status (yes/no) and grade of iENE (1–3).

#### Definition and grading of iENE

Criteria for defining iENE were agreed according to the International Collaboration of Oropharyngeal Cancer Network (ICON-N) scale [14] and international consensus recommendations from the Head and Neck Cancer International Group (HN-CIG) [15].

**Fig. 1:**
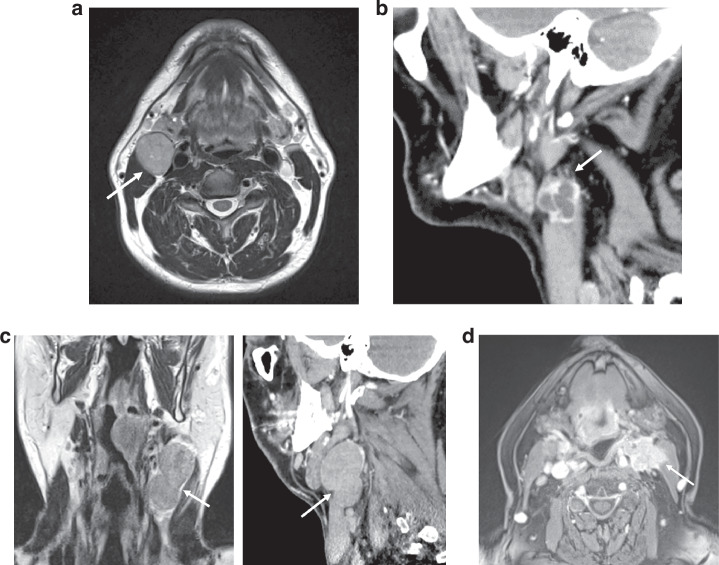
4-point scale used to grade iENE. **a** Grade 0 iENE: Axial T2 MRI. Well-defined metastatic Level II node, clear perinodal fat (arrow). **b** Grade 1 iENE: Sagittal CECT. Metastatic Level II node with an irregular nodal margin + perinodal fat stranding (arrow). **c** Grade 2 iENE: Coronal T2 MRI + sagittal CECT demonstrating coalescence of metastatic Level II + III nodes (arrow). **d** Grade 3 iENE: Axial T1FS+c MRI demonstrating definite invasion of a metastatic Level II node into adjacent structures. Note the direct infiltration of sternocleidomastoid muscle (arrow)

The 4-point scale used to score iENE [14, 15] is illustrated in the examples in Fig. 1a–d:

- Grade 0: No iENE
- Grade 1: Irregular or ill-defined nodal margins and /or definite extension into perinodal fat.
- Grade 2: Coalescent nodes. Nodal fusion with loss of the intervening nodal capsular and perinodal fat planes on multiplanar assessment.
- Grade 3: Definite extension into adjacent structures such as muscle, skin, glands, neurovascular bundle.

As per published literature [15], the most suspicious node was graded in each case and the nodal level of that node was annotated (for correlation with pathology report). Other neck nodes were not assessed.

#### Protocol for imaging review

MRI and CT staging studies were performed using different MRI & CT systems across the three centres but there were no major differences in the CT & MRI protocols utilised in the different centres. Image quality was assessed for each case and deemed to be suboptimal if any of the following criteria applied:

- Intravenous contrast enhanced CT/MRI not performed
- Slice thickness >3 mm
- Significant motion degradation affecting nodal assessment
- Multiplanar sequences not obtained

Ultrasound (US) was performed as standard of care in the majority of cases but was not included in the iENE assessment.

#### First round reviews (pre-consolidation)

291 cases were independently scored by two radiologists blinded to the score of the other. Discordant cases were identified for re-review.

#### Consolidation

**Fig. 2:**
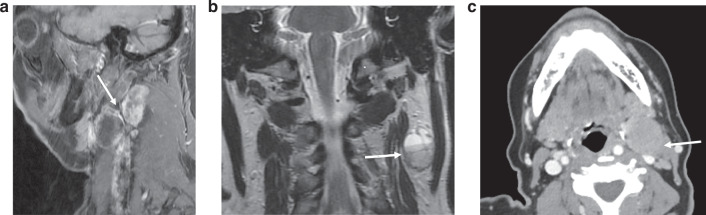
Principles to promote inter-rater agreement in iENE assessment. **a** Grade 1 iENE: Sagittal T1FS+c MRI– showing two adjacent metastatic Level II + III nodes with irregular nodal margins and perinodal fat stranding, but with a clear fat plane separating the 2 nodes. These nodes had appeared to be coalescent on axial images but review of coronal + sagittal imaging planes resulted in Grade 1 iENE. **b** Grade 0 iENE: Coronal T2 MRI showing cortical lobulation within a cystic metastatic Level II node (arrow), lobulation of a single metastatic node should be should be distinguished from coalescence of adjacent nodes to avoid overcalling Grade 2 iENE. This node has a well-defined margin and there is no perinodal fat stranding, therefore Grade 0 iENE. **c** Grade 0 iENE: Axial CECT demonstrating loss of the fat plane between a metastatic Level II node and sternocleidomastoid muscle (arrow). However, there is no extranodal enhancement to indicate muscle invasion of the muscle (compare with Fig. 2d), therefore Grade 0 iENE.

The broad patterns of disagreement after first reading as shown in Table 1 were shared with the 4 radiologists. This highlighted one major area of disagreement: where one radiologist scored iENE 0 and the other scored iENE=1. The radiologists met to review and address these data, share their experiences, review the iENE assessment criteria, and collectively review several non-study cases to promote inter-rater concordance in iENE assessment—a method that has been used by others [14]. The following principles were agreed upon, illustrated in the examples in Fig. 2a–c:

- Only *unequivocal* iENE (of any grade) should be scored, visible on all planes and sequences
- Fat stranding should be unequivocal on all sequences for grade 1 iENE
- A coalescent nodal mass should be unequivocal on all sequences for grade 2 iENE
- Loss of fat plane with an adjacent structure (e.g sternocleidomastoid) should only be scored as grade 3 iENE if accompanied by invasion of the adjacent structure.
- Lobulation of a single lymph node should not be considered grade 2 iENE
- Eccentric cortical hypertrophy should not be considered grade 2 iENE
- Any iENE feature interpreted as iatrogenic (e.g. recent core biopsy) should not be scored as iENE.

**Table 1:** Baseline characteristics.

		N = 291
**Demographics**		
**Sex**	Male	234 (80.4)
	Female	57 (19.6)
**Age (years)**	Median (IQR)	57.7 (52.5–63.6)
**Smoking history**	Never	151 (51.9)
	Current/Ex	140 (48.1)
**Clinical**		
**cT category**	T1	130 (44.7)
(TNMv7)	T2	151 (51.9)
	T3	10 (3.4)
**cN category**	N0	32 (11.0)
(TNMv7)	N1	68 (23.4)
	N2a	64 (22.0)
	N2b	127 (43.6)
**Side of primary tumour**	Left	133 (45.7)
	Right	156 (53.6)
	Central	1 (0.3)
	Missing	1 (0.3)
**Highest ipsilateral nodal level**	I	4 (1.4)
	II	184 (63.2)
	III	52 (17.9)
	IV	21 (7.2)
	V	5 (1.7)
	No involved levels	24 (8.3)
	Missing	1 (0.3)
**Highest contralateral nodal level**	II	2 (0.7)
	No involved levels	288 (99.0)
	Missing	1 (0.3)
**Anatomical site**	Tonsil	192 (65.9)
	Soft palate	1 (0.3)
	Tongue base/ Vallecula	66 (22.7)
	More than one	31 (10.7)
	Missing	1 (0.3)
**Pathological**		
**pT category**	TX	5 (1.7)
(pTNMv7)	T1	134 (46.1)
	T2	133 (45.7)
	T3	17 (5.8)
	T4a	2 (0.7)
**pN category**	NX	2 (0.7)
(pTNMv7)	N0	31 (10.7)
	N1	66 (22.7)
	N2a	68 (23.4)
	N2b	120 (41.2)
	N3	4 (1.4)
**TNM stage 7**	I	8 (2.8)
	II	18 (6.2)
	III	66 (22.7)
	IVA	190 (65.3)
	IVB	4 (1.4)
	Missing	5 (1.7)
**Number of lymph nodes identified**	0	2 (0.7)
	1–10	12 (4.1)
	11–20	97 (33.3)
	21–30	96 (33.0)
	>30	83 (28.5)
	Missing	1 (0.3)
**Number of positive lymph nodes**	0	29 (10.0)
	1	123 (42.3)
	2	70 (24.1)
	3	39 (13.4)
	4+	26 (8.9)
	Missing	4 (1.4)
**Presence of pathological extranodal extension (pENE)**	Yes	212 (72.9)
	No	79 (27.2)
**Radiological**		
**MRI available**	Yes	260 (89.4)
	No	31 (10.7)
**Sub-optimal imaging**	Yes	54 (18.6)
	No	236 (81.1)
	Uncertain	1 (0.3)
**Core biopsy prior to MRI (or CT if no MRI)**	Yes	111 (38.1)
	No	180 (61.9)
**If yes, no of days**	0–10	65 (58.6)
	11–30	34 (30.6)
	31–50	9 (8.1)
	50–73	3 (2.7)

#### Second round reviews (post-consolidation)

Discordant cases were re-reviewed and independently scored by the two radiologists who had initially reviewed the cases, blinded again to the score of the other. Cases with persistent discordance after 2 rounds were reviewed together to reach a consensus score. For cases where agreement could not be reached, ‘arbitration’ by radiologists from the other team was required.

#### QA audit across both teams of radiologists

In order to ensure consistency in iENE assessment between the two teams of radiologists, 40 cases (20 per team), representing approximately 14% of cases overall, were randomly selected, anonymised and uploaded into a DICOM web viewer (http://get.pacsbin.com/) for review by the other team. The results were analysed for inter-team agreement.

### Pathological Extranodal Extension (pENE) assessment

**Fig. 3:**
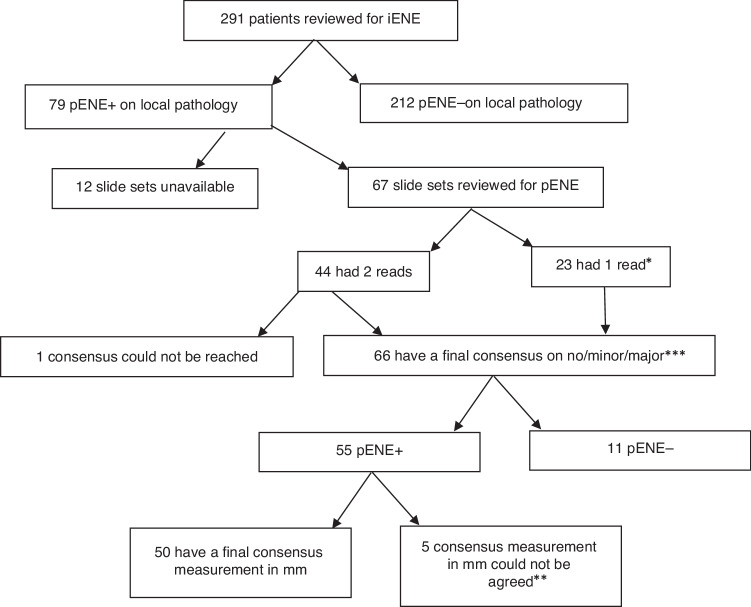
Flow diagram of iENE and pENE review. *One reader only checked with another reader when uncertain. **Five cases with “major” pENE could not have measurements in mm agreed. ***55 had pENE after review by the study pathologists.

The 291 cases included in this sub-study had all undergone surgery and pathology assessment at their local hospital, in accordance with the PATHOS protocol. Of these 291 cases, 79 cases had pENE recorded on local pathology reports entered into the trial pathology Case Report Forms (CRFs), 12 of which had missing slide sets (see Fig. 3). The pathology slides of the remaining 67 cases were reviewed by six specialist head and neck pathologists at Newcastle (2), Liverpool (2) and South-East Wales (2). Histology slides from the remaining 212 cases, without evidence of pENE entered into the trial CRFs, were not reviewed as comprehensive pathology QA review of surgical specimens from PATHOS phase II had shown high concordance in pENE reporting between local site pathologists and the central trial pathology QA team (only 1/83 (1.2%) local pENE- cases and 3/39 (7.7%) local pENE+ cases were discrepant, overall concordance 118/122 = 96.7%).

#### Definition and measurement of pENE

Criteria for defining pENE were agreed according to international consensus recommendations from the HN-CIG [16]. Categorisation into minor/microscopic pENE (≤2 mm) and major (>2 mm) pENE, as well as measurement of pENE in mm from the nodal capsule were recorded.

#### First round reviews

67 cases were divided between three pathology teams; most cases were independently reviewed and scored by two pathologists who were blinded to the assessment of the other; in one centre both pathologists reviewed all cases together.

#### Re-review of discordant cases

Where discordant cases were identified, both pathologists from a centre met to review and discuss the cases together and, where possible, come to consensus.

#### Arbitration

Where there was persistent uncertainty or disagreement between pathologists from a centre, ‘arbitration’ was carried out by a pathologist from another centre.

### Statistical analysis

All analyses were pre-specified in a statistical analysis plan and conducted using Stata 18. We compared inter-observer variability for iENE and pENE using observed agreement and Gwet’s agreement coefficient (AC1) which adjusts for chance agreement due to category prevalence [17]. We calculated sensitivity and specificity of iENE for pENE and interrater concordance by Gwet’s AC1 score using different cutoff scores for iENE and pENE. Cases missing pENE score were excluded from the analysis. The sample size of 291 patients (with 79 having pENE) enabled us to calculate sensitivity of iENE with 95% confidence intervals of width 22% at most and specificity with 95% confidence intervals of width 14% at most. Gwet’s AC statistic was interpreted using benchmark scales of Landis and Koch [18, 19], ranging from poor agreement (0.00–0.20) to very good agreement (0.81–1.00).

## Results

### Clinical characteristics of the study population

The first 291 patients consecutively recruited into the PATHOS trial from Liverpool (*n *= 163), Newcastle (*n* = 79) and South-East Wales (*n* = 49) were included. Table 1 shows their baseline characteristics: median age was 57.7 years (IQR: 52.5-63.6), 234/291 (80.4%) were male, 289/291 (99%) had tonsil/tongue base or overlapping primaries, 281/291 (96.6%) had cT1-2 disease, and 259/291 (89%) had ipsilateral nodal disease (TNMV7 category N1, N2a, N2b). On pathology specimens, 79/291 (27.2%) had evidence of pENE noted on local pathology reports. Median time from staging scans to surgery was 39 days (IQR: 27-54, range: 6-94).

### Inter-rater agreement for detection of imaging-detected Extranodal Extension (iENE)

#### Round 1

Table 2 shows the inter-rater agreement between the 1st and 2nd reader for iENE after the first read. Readers agreed the same iENE score on the 4-point scale (from 0 to 3) for 142/291 (0.49 (95% CI: 0.43–0.55)) of cases (Gwet’s AC1: 0.34 (95%CI: 0.26–0.41)). Of the 149 discordant cases, 107 (71.8%) occurred when one reader scored 0 and one reader scored >0 i.e. whether or not any imaging features of iENE were present. Compared to four iENE categories, agreement, but not Gwet’s AC1, was significantly higher if iENE was dichotomised on 0 (no iENE) versus grade 1/2/3 iENE+ (Agreement: 0.63 (95% CI: 0.58–0.69), Gwet’s AC1: 0.29 (95% CI:0.17–0.40)). Compared to four iENE categories, both agreement and Gwet’s AC1 were significantly higher if iENE was dichotomised on 0-1 versus grade 2/3 iENE+ (Agreement: 0.76 (95%CI:0.71-0.81), Gwet’s AC1: 0.55 (95%CI: 0.45-0.65). The same trends were seen in each reader team subgroup (Supplementary Tables S1 and S2).

**Table 2:** Inter-rater agreement of iENE.

First reading								
Cases						Agreement measures		
	**Reader 2 – iENE score**							
	**0**	**1**	**2**	**3**	**Total**		**Agreement (95%CI)**	**Gwet’s AC (95%CI)**
**Reader 1 – iENE score**								
**0**	65	61	39	1	**166**	4 categories	0.49 (0.43–0.55)	0.34 (0.26–0.41)
**1**	3	22	7	3	**35**	Binary: 0 vs 1/2/3	0.63 (0.58–0.69)	0.29 (0.17–0.40)
**2**	1	14	52	10	**77**	Binary: 0/1 vs 2/3	0.76 (0.71–0.81)	0.55 (0.45–0.65)
**3**	2	4	4	3	**13**			
**Total**	**71**	**101**	**102**	**17**	**291**			

Second reading								
Cases						Agreement measures		
	**Reader 2 – iENE score**							
	**0**	**1**	**2**	**3**	**Total**		**Agreement (95%CI)**	**Gwet’s AC (95%CI)**
**Reader 1 – iENE score**								
**0**	173	5	10	0	**188**	4 categories	0.91 (0.87–0.94)	0.89 (0.85–0.93)
**1**	5	26	1	0	**32**	Binary: 0 vs 1/2/3	0.92 (0.89–0.95)	0.85 (0.79–0.91)
**2**	3	1	62	1	**67**	Binary: 0/1 vs 2/3	0.95 (0.92–0.97)	0.91 (0.87–0.96)
**3**	**0**	**1**	**0**	**3**	**4**			
**Total**	**181**	**33**	**73**	**4**	**291**			

#### Round 2

Table 2 shows the inter-rater agreement between the 1st and 2nd reader for iENE after the second read. Agreement was high (>0.9), as was Gwet’s AC1 (≥0.85), for all three categorisations of iENE, with agreement scores significantly higher for the 2nd read and Gwet’s AC1 scores also significantly higher, changing from fair/moderate to very good agreement. The same trends were seen in each reader team subgroup (Supplementary Tables S1 and S2). A minor improvement was seen when only those cases with MRI imaging available were included (Supplementary Table S3).

There remained disagreement on iENE score for 27 cases after second read; consensus was reached through discussion within teams on 20 of these cases, but one team could not reach consensus on 7 cases which were passed to the other team for arbitration—all 7 cases were scored as 0 (no “unequivocal” evidence of iENE).

Overall, pre-operative cross-sectional imaging (CT+/− MRI) was scored for iENE grade (0-3) for 291 cases: 96/291 (33.0%) were positive (grade 1-3) for iENE after 2nd round reviews and consensus.

#### QA audit across both teams of radiologists

A random 20 cases from each team were reviewed by the other team (one case could not be uploaded for review). Although concordance was high within teams (18 out of 19 (94.7%) cases in one team and 19 out of 20 (95.0%) cases in the other) there remained some discordance between teams (agreement on 15 out of 19 (78.9%) between team 1 and 2 and 16 out of 20 (80.0%) between team 2 and team 1). None of the discrepancies could be attributed to suboptimal imaging.

### Inter-rater agreement for detection of pathological Extranodal Extension (pENE)

Figure 3 shows the flow of slides through the pENE review process. 67 patients with pENE noted by local pathologists had slides reviewed by central pathologists, 44 of which were read independently by a first and a second reader. Supplementary Table S4 shows the inter-rater agreement between the 1st and 2nd reader—readers agreed the same score for 33/44 (Agreement: 0.75 (95%CI: 0.62–0.88), Gwet’s AC1: 0.69 (95% CI: 0.51–0.87)). Agreement was higher if pENE was dichotomised on none versus minor/major: 40/44 (Agreement=0.91, 95%CI: 0.82–1.00; Gwet’s AC1 = 0.90, 95%CI: 0.79–1.00). After re-review, consensus could not be reached for 1 case, so it was excluded from the analyses. 66 had final consensus agreement on no/minor/major pENE (55 pENE+ and 11 pENE-), 50 of the pENE+ also had final consensus agreement on measurement in mm (measurement could not be agreed for 5 cases).

### Correlation between iENE and pENE

278 patients were available for correlation between pENE and iENE (Fig. 3: 12 slide sets could not be traced and for one patient presence/absence of pENE could not be agreed). As shown in Table 3, 31 out of 55 (56.4%) cases were true positive of iENE>0 for pENE+ and 165 out of 223 (74.0%) were true negative.

**Table 3:** Agreement between iENE and pENE.

		pENE divisions of none/minor/major (numbers are numbers of cases)							
		None	Minor (0–2 mm)		Major (>2 mm)				Total
**iENE**	**0**	165	6		18				189
	**1**	19	1		6				26
	**2**	38	5		17				60
	**3**	1	1		1				3
	**Total**	**223**	**13**		**42**				278***

		pENE divisions of mm from nodal capsule (numbers are numbers of cases)							
		None	>0 to ≤ 1 mm	>1 to ≤2 mm	>2 to ≤ 3 mm	>3 to≤4 mm	>4 to ≤ 5 mm	>5 mm	Total
**iENE**	**0**	165	0	6	9	3	2	4	189
	**1**	19	1	0	2	2	0	1	25
	**2**	38	4	1	3	3	0	7	56
	**3**	1	1	0	0	0	1	0	3
	**Total**	223	6	7	14	8	3	12	273***

			pENE: extension of tumour beyond nodal capsule						
			>0 mm	>1 mm	>2 mm	>3 mm	>4 mm	>5 mm (n = 12)	
	n*		55	49	42	23	15	12	
	N**		278	278	278	273***	273***	273***	
**iENE**	**0 or 1/2/3**	Agreement	0.71 (0.65–0.76)	0.68 (0.63–0.74)	0.70 (0.65–0.76)	0.71 (0.66–0.76)	0.70 (0.65–0.76)	0.71 (0.65–0.76)	
		Gwet’s AC	0.52 (0.42–0.63)	0.50 (0.39–0.60)	0.53 (0.43–0.64)	0.58 (0.48–0.68)	0.58 (0.48–0.68)	0.59 (0.49–0.68)	
		Sensitivity	56.4 (42.3–69.7)	51.0 (36.3–65.6)	57.1 (41.0–72.2)	60.9 (38.5–80.3)	60.0 (32.2–83.7)	66.7 (34.9–90.1)	
		Specificity	74.0 (67.7–79.6)	72.1 (65.8–77.8)	72.5 (66.3–78.1)	72.0 (66.0–77.5)	70.9 (65.0–76.4)	70.9 (65.0–76.3)	
	**0/1 or 2/3**	Agreement	0.75 (0.70–0.80)	0.73 (0.68–0.79)	0.75 (0.70–0.80)	0.78 (0.73–0.83)	0.79 (0.74–0.84)	0.79 (0.74–0.84)	
		Gwet’s AC	0.62 (0.53–0.71)	0.61 (0.51–0.70)	0.64 (0.55–0.73)	0.70 (0.63–0.78)	0.72 (0.65–0.80)	0.73 (0.66–0.81)	
		Sensitivity	43.6 (30.3–57.7)	38.8 (25.2–53.8)	42.9 (27.8–59.0)	47.8 (26.8–69.4)	53.3 (26.6–78.7)	58.3 (27.7–84.8)	
		Specificity	82.5 (76.9–87.2)	80.8 (75.1–85.7)	80.9 (75.3–85.7)	80.8 (75.4–85.5)	80.2 (74.8–84.9)	80.0 (74.7–84.7)	

*number fulfilling the pENE measurement.

**number of cases available for calculation of agreement measures.

***5 “major pENE” did not have measurements.

NB. 2 soft tissue metastases (STMs) treated as >5mm.

Example calculations for clarity:

For iENE 0 or 1/2/3 and pENE 0 or >0mm: sensitivity=TP/(TP+FN)=(1+6+5+17+1+1)/ (1+6+5+17+1+1+6+18)=56.4%; specificity=TN/(TN+FP)=165/(165+19+38+1)=74.0%.

For iENE 0/1 or 2/3 and pENE 0 or >0mm: sensitivity=TP/(TP+FN)=(5+17+1+1)/ (5+17+1+1+6+18+1+6)=43.6%; specificity=TN/(TN+FP)=(165+19)/(165+19+38+1)=82.5%.

For iENE 0 or 1/2/3 and pENE ≤5mm or >5mm: sensitivity=TP/(TP+FN)=(1+7)/ (1+7+4)=66.7%; specificity=TN/(TN+FP)=(165+6+9+3+2)/(165+6+9+3+2+19+1+2+2+38+4+1+3+3+1+1+1)=70.9%.

For iENE 0/1 or 2/3 and pENE ≤4mm or >4mm: sensitivity=TP/(TP+FN)=(1+7)/ (1+7+2+4+1)= 53.3%; specificity=TN/(TN+FP)=(165+6+9+3+19+1+2+2)/(165+6+9+3+19+1+2+2+38+4+1+3+3+1+1)=80.2%.

Table 3 shows the agreement between iENE (showing both grade 1/2/3 as iENE+ and grade 2/3 as iENE+) and pENE (dichotomised as different extensions of tumour beyond the nodal capsule). For iENE predicting any pENE, there was a trend for higher sensitivity and lower specificity when a lower grading threshold for iENE was used (i.e. 1/2/3 as iENE+ rather than grade 2/3 as iENE+) (sensitivity 56.4% (95%CI: 42.3–69.7) versus 43.6% (95%CI:30.3–57.7) and specificity 74.0% (95%CI: 67.7–79.6) versus 82.5% (95%CI: 76.9–87.2) respectively) and this trend was true for all extensions of pENE. There is a trend for increasing sensitivity and decreasing specificity, as pENE is defined by increasing extension of the tumour beyond the nodal capsule. Regardless of cut-offs used, sensitivity of iENE for pENE was low (at best: 56.4% (95%CI: 42.3–69.7) and specificity high (at worst: 70.9% (95%CI: 65.0–76.3)).

### Impact of image quality and core biopsy on iENE assessment

54 (18.6%) of 291 patient staging CT or MRI scans (Table 1) had suboptimal image quality for iENE assessment, with optimal imaging quality rates varying from 68.7% to 98.7% across the different centres. Adequate image quality was lower at one trial site (68.7%) to the other two (>90% adequacy). This was not primarily due to MRI/CT hardware but related to MRI study technique. Inadequate MRI quality with motion-degraded images and thicker slices was the main reason for inadequate quality. Furthermore, 111/291 (38.1%) cases had a core biopsy <30 days prior to cross-sectional imaging. A sensitivity analysis was conducted to assess the impact of removing these cases from the correlation between pENE and iENE (Supplementary Table S5); modest improvements were seen in sensitivity (up to 59.4% (95% CI: 40.6-76.3) for 1/2/3 as iENE + ) and specificity (up to 87.8% (95%CI: 80.4-93.2) for 2/3 as iENE + ).

### Discordant case reviews

#### iENE+/pENE- (false positive) cases

As shown in Table 3, iENE was identified on imaging in 19 + 38 + 1 = 58 (26.0%) of the 223 cases with no pENE documented on their post-operative pathology CRFs (iENE + /pENE- cases i.e. false positives). 22 of these had been confirmed as having no pENE during the PATHOS trial pathology comprehensive QA review process; histology reports for the remaining discordant cases were reviewed by the study pathologists who confirmed that no cases of pENE had been missed from the CRF. Two recurring features were recorded on the histology reports in 16/36 (44.4%) cases which could potentially mimic iENE on imaging:

- Node(s) associated with marked perinodal fibrosis/perinodal fibroplasia noted in 12/36 (33.3%) cases; the mean diameter of metastatic nodes associated with perinodal fibrosis was 33.1 mm (range 19–59 mm): 8 of these were iENE=2 and 4 were iENE=1
- A “conglomerate nodal mass”, or “at least 2 fused nodes”, or “matted nodes” in 4/36 (11.1%) cases: 2 were iENE=2 and 1 was iENE=1.

Supplementary Table S6 shows the cases with suboptimal imaging and core biopsy prior to imaging in iENE/pENE discordant and concordant cases. It can be seen that the proportion of cases with sub-optimal imaging was highest for iENE + /pENE- cases (17/58 (29.3)).

#### iENE-/pENE+ (false negative) cases

As shown in Table 3, iENE was not identified on imaging in 6 + 18 = 24 (40.0%) of the 55 cases with pENE (iENE-/pENE+ cases i.e. false negatives). Six of these were minor and 18 major pENE. The gap between staging and surgery was the same in this subgroup of *n* = 24 patients (median 36 days, IQR: 22-55, range: 3–74) as for the whole cohort.

## Discussion

In PATHOS-ENE, we have utilised the PATHOS trial to interrogate the correlation between imaging-detected and pathological extranodal extension (ENE) in patients with HPV-positive oropharyngeal cancer (OPSCC). This correlation can only be explored in a surgically treated cohort of patients, and PATHOS-ENE is the first study aligned to a prospective multi-centre clinical trial to conduct these analyses. By reviewing the first 291 participants recruited to PATHOS at three regional UK cancer centres, we demonstrated that:

i) The sensitivity of iENE for predicting pENE was relatively low (at best: 56.4% (95%CI: 42.3–69.7) and the specificity was high (at worst: 70.9% (95% CI: 65.0–76.3)).
ii) There was a trend for higher sensitivity and lower specificity of iENE for predicting pENE when all grades (1, 2 and 3) of iENE were included compared to a higher threshold (grades 2 and 3 only).
iii) Excluding cases with suboptimal image quality and recent core biopsy produced modest improvements in sensitivity (up to 59.4% (95% CI: 40.6–76.3) for 1/2/3 as iENE+) and specificity (up to 87.8% (95%CI: 80.4–93.2) for 2/3 as iENE+).
iv) Inter-rater variability exists in the assessment of both iENE and pENE, and inter-rater agreement is improved by shared experience and consensus building. After 2nd review, our radiologists agreed the same score from (0–4) for iENE in 264/291 (0.91) of cases. In a QA audit of 20 cases, radiologists from different teams had higher discordance for iENE assessment than those in the same team, suggesting that working together improves inter-rater agreement.

Pathological extranodal extension (pENE) is defined by the College of American Pathologists as “extension of metastatic tumour, present within the confines of the lymph node, through the lymph node capsule into the surrounding connective tissue, with or without associated stromal reaction” and is measured from the external aspect of the lymph node capsule to the most distant tumour focus. Generally, pENE is regarded as a poor prognostic factor in HNSCC, correlating with increased risk of regional and distant recurrence, reduced overall survival and an indication for adjuvant chemo-radiotherapy, based on the results of two landmark studies, RTOG 9501 and EORTC 22931 [5–7]. The impact of pENE on prognosis from HPV-positive OPSCC was considered less significant, based on the results of several studies that informed the AJCC/UICC V8 pathological staging classification of HPV-positive OPSCC [20]. However, recent data demonstrate that pENE does adversely affect prognosis in HPV-positive OPSCC in keeping with other HNSCCs [4].

Imaging-detected extranodal extension (iENE) has also been shown to adversely affect prognosis for HPV-positive OPSCC [3, 6, 7] and other locally advanced head and neck squamous cell carcinomas [21], and proposals to include iENE, as well as pENE, in an updated staging classification for HPV-positive OPSCC are being developed by the AJCC and UICC. To date, studies of iENE have predominantly included patients treated with primary radiotherapy/chemo-radiotherapy, and they have not been able to correlate the diagnosis of iENE with pathology. Conversely, studies of pENE have, by necessity, included patients who undergo neck dissection, and have not until recently correlated pENE with iENE status on pre-operative imaging. Whilst the impact of iENE and pENE on prognosis may be independent of the correlation between them, understanding this relationship is important for many reasons, including multi-disciplinary treatment decision-making. The presence or absence of iENE is often used to direct clinical decision-making. When present, patients will be offered primary chemo-radiotherapy, because if such patients had surgery and pENE was subsequently confirmed on histology, they would have to be offered post-operative chemo-radiotherapy in addition to surgery. This ‘triple modality therapy’ is unnecessary and potentially harmful as existing data confirm that chemo-radiotherapy alone amounts to appropriate standard of care treatment. Understanding the correlation between iENE and pENE, and therefore the accuracy of iENE in predicting the presence or absence of pENE, is vital when recommending treatment, specifically when surgery is to be offered or not. Our data confirm that the sensitivity of iENE to predict pENE is relatively low (low true positive rate) and therefore it is not recommended as a means to triage patients away from surgery. In contrast, our data confirm a high iENE specificity (high true negative rate) providing confidence that in the absence of iENE there is a low probability of pENE if surgery was to be offered.

In our cohort, there were 58 iENE+/pENE- cases (false positives), of which 36 were reviewed for pENE. Interestingly, 44% had either perinodal fibrosis (33.3%) or a conglomerate nodal mass (11.1%) on histology. The frequency of these features may indeed be higher than reported here, as a lack of reference to them on the histology report is not synonymous with absence. Nevertheless, the suggestion from these data that perinodal fibrosis/fibroplasia may mimic ENE during the radiological staging of HPV-positive OPSCC is tantalising and warrants further investigation. Furthermore, the finding that matted nodes and/or a conglomerate nodal mass on histology does not automatically represent pENE if the nodal capsule(s) remain intact (without tumour extension between nodes), has implications for radiological nodal assessment. Grade 2 iENE should only be scored if ≥2 adjacent lymph nodes have lost their intervening tissue planes and capsules to form a single indivisible structure.

24 iENE-/pENE+ cases (false negatives) were also identified. Tumour progression between staging and surgical resection may be a factor in these cases. The PATHOS protocol mandates surgery within 6 weeks of registration, and the median interval between registration and surgery in the whole trial is 8 (IQR: 1-10) days. Staging investigations, including cross-sectional imaging, must be carried out within 10 weeks of study entry. The interval between staging scans and surgery for the 278 cases in the correlative PATHOS-ENE sub-study was 39 days (IQR: 27-54, range 6-94) and was similar in the 24 iENE-/pENE+ cases (median 36 days, IQR: 22-55, range: 3-74), suggesting that tumour progression was not a major factor in false negative cases.

**Fig. 4:**
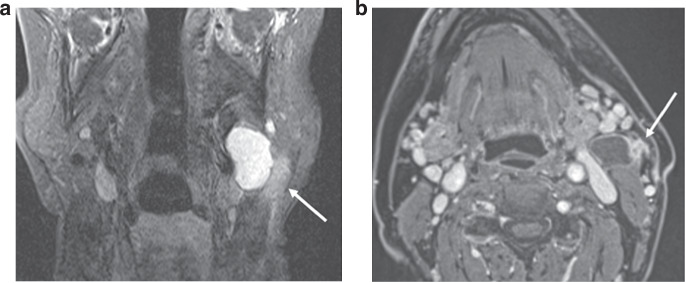
Iatrogenic changes post core biopsy—example 1. HPV-positive OPSCC, 6 days following core biopsy of a cystic left Level II node. **a** Coronal STIR MRI**; b** Axial T1FS+c MRI, showing minor localised perinodal stranding and enhancement at the lateral margin of the node (arrows). The position corresponds to the core biopsy needle path.

**Fig. 5:**
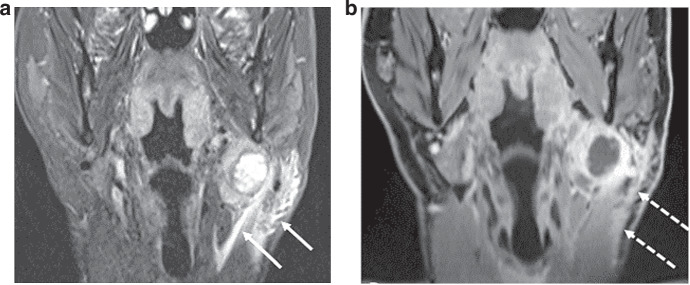
Iatrogenic changes post core biopsy—example 2. HPV-positive OPSCC 1 day following core biopsy of a necrotic left Level II node. Images show: **a** Coronal STIR MRI; **b** Coronal FS T1+c MRI, showing extensive post-biopsy oedema and stranding in the left upper neck (arrows). Note also the platysma muscle thickening and hyperenhancement (dotted arrows).

iENE assessment is highly dependent on good quality imaging and we found significant heterogeneity in image quality (especially MRI) in the PATHOS cohort, with 18.6% of images acquired over the 9-year recruitment period being deemed suboptimal. The two centres where staging scans were performed in a small number of centralised, high-volume sites had a significantly lower rate of suboptimal imaging than the third centre where imaging was performed across a larger number of referral sites. Factors affecting image quality include post-contrast slice thickness and multiplanar image analysis. These differences are rarely due to different MRI or CT protocols but rather a combination of factors including system quality, radiographic technique and radiologist supervision. Major pENE, defined as >2 mm ENE, may not be reliably detected with imaging slice thickness ≥3 mm due to partial volume artefact (averaging of signal from adjacent small structures into single voxel), and minor pENE ( ≤ 2 mm) will be missed altogether. Furthermore, as lymph nodes in the deep cervical chain are aligned longitudinally along the jugular vein, nodal coalescence will tend to occur along this axis, thus multiplanar assessment of both coronal and sagittal images is needed to accurately identify nodal coalescence (iENE grade 2). Iatrogenic changes are another confounder and 38% of study cases (111 patients) underwent a core nodal biopsy prior to their staging CT and/or MRI scans. Knowing if and when a core biopsy has been carried out is imperative before cross-sectional imaging is reviewed for iENE, as iatrogenic biopsy effects can vary from minimal perinodal fat-stranding to changes that mimic grade 3 iENE, examples of which are shown in Figs. 4 and 5. Following the 1st round of radiology reviews, it was agreed that any potential biopsy-related changes should be discounted and not recorded as iENE for the 2nd (final) round reviews, following general TNM principles (14). This obviously has a potential for producing false negative results where genuine iENE features are masked by iatrogenic change and we saw the highest proportion of recent (<30 day) core biopsies in the iENE-/pENE+ group. Conversely core biopsy may also produce false positives if biopsy related findings are interpreted as positive iENE features, particularly if the reporting radiologist is unaware of recent biopsy (Figs. 4 and 5).

Although review of ultrasound (US) imaging was not included for iENE assessment in this study, neck US is often the first-line neck lump investigation in the UK, and US-guided core biopsy is often carried out before any cross-sectional imaging is performed. While the superior spatial resolution of US makes it ideal for detecting features of iENE *pre*-biopsy, there is far less published literature on the accuracy of iENE assessment by US than by cross-sectional imaging modalities. Prospective research is required to assess whether neck US is indeed the optimal patient encounter for iENE assessment.

Intuitively, we would expect cases with smaller pENE extensions to be more frequently recorded as iENE negative but, as shown in Table 3 this was not the case. Of the 24 discordant cases, 4 had pENE >5 mm from nodal capsule, and 3 of these 4 cases had prior core biopsies (done 6, 11 and 19 days prior to cross-sectional imaging). Classification of pENE into minor/microscopic ENE (≤2 mm) or major (>2 mm) ENE is recommended by the AJCC for data collection and future analysis. However, the prognostic significance of minor vs major ENE is unclear for all HNSCCs and is complicated by the varying classifications used in the literature, the subjective nature of its assessment, and the effect of adjuvant treatment. In our study, there was a numerical trend for better agreement and sensitivity with iENE as the pENE cut off increased from >0 mm to >5 mm, and the correlation was greatest for pENE extensions >5 mm.

A 1 mm pENE cut-off has become increasingly important in the context of surgically treated HPV-positive OPSCC as a result of the ECOG-ACRIN 3311 phase II RCT that classified patients with ≤1 mm pENE on post-operative histology as intermediate risk (to receive adjuvant radiotherapy only) and >1 mm pENE as high-risk (to receive adjuvant chemo-radiotherapy) [22]. Table 3 shows that no value of iENE selects well for those who are pENE >0 and ≤1 mm. Therefore, iENE cannot be used to accurately identify patients with minor (≤1 mm) pENE pre-operatively and iENE should not be used as a parameter to automatically triage patients away from undergoing surgery. This is particularly relevant as the excellent outcomes reported in ECOG 3311 Group B participants (a proportion of whom will have ≤1 mm pENE), suggest that avoidance of adjuvant chemo-radiotherapy is a realistic future prospect for patients with ≤1 mm pENE. Once the primary endpoint of the PATHOS trial matures, we will be able to further establish the prognostic significance of pENE (and/or iENE) for HPV-positive OPSCC and the benefit, or otherwise, of adjuvant chemo-radiotherapy when pENE is present.

It is important to highlight that the influence of iENE on prognosis in HPV-positive OPSCC has been demonstrated independently of its correlation with pENE [3, 8]. Our results show that in the PATHOS cohort, a diagnosis of iENE is not always predictive of pENE, a finding that is consistent with a recently published retrospective study of patients treated with surgery and/or chemo-radiotherapy in the ‘real world’ setting [23]. It is likely that iENE and pENE will never match completely, raising the possibility that iENE might be detecting something extra (e.g. nodal volume, nodal burden and/or an as yet unidentified phenomenon) that is a useful marker of disease behaviour and clinical outcome.

We have demonstrated inter-observer variability in the assessment of iENE and pENE, in keeping with previously published literature [14, 24–26], and that there is a ‘learning curve’ that can be overcome, particularly for iENE, by sharing experiences and expertise, consolidating definitions and assessment methods, and coming to consensus on when to score certain findings on imaging. Recently published international guidelines and their accompanying atlases can certainly help navigate this learning curve [15, 16]. An important principle of staging is that in cases where there is doubt, a patient should be down-staged, rather than up-staged. As iENE and pENE look set to be adopted into the next version of the TNM staging classification for HPV-positive OPSCC, it is critical that they are only diagnosed when there is a high degree of certainty that they are indeed present – as such, only ‘unequivocal’ iENE (regardless of grade) and ‘definitive’ pENE (regardless of major/minor, or measurement in mm) should be reported. The principle of “not missing any ENE” is trumped by the importance of “preserving the prognostic value of ENE”. As well as clear guidance, high-quality training sets/materials will need to be accessible to the head and neck community globally to enable ENE to be incorporated in a standardised way into future staging systems for HPV-positive OPSCC. It should also be noted that we found a discordance of 11/66 (16.7%) in the local pENE+ cases during the central review undertaken in this PATHOS-ENE sub-study; this was higher than the 7.7% in the trial QA, possibly because central review for the sub-study involved multiple pathologists rather than just one as in the trial QA. Furthermore, until recently [16], there has been no consensus on the diagnostic criteria, interpretation, and reporting of pathological extranodal extension which contributes to clinical inconsistency.

This PATHOS-ENE sub-study has many strengths, including the inclusion of a homogenous cohort of patients with HPV-positive OPSCC, all with well-annotated, prospectively collected baseline and post-operative pathological data. Over 65% of patients included had TNMV7 N2 disease, and 27.2% had pENE on their post-operative histology, which is consistent with pENE rates in previous surgical cohorts [27]. One caveat of studying the correlation between iENE and pENE in the PATHOS cohort is that cases of clinically overt ENE and/or Grade 3 iENE may have been excluded from enrolment in participating centres wanting to avoid Group C allocation and randomisation to adjuvant chemo-radiotherapy. Data supporting this include the low incidence of grade 3 iENE (1%) in our study population, which contrasts with another study of patients with locally advanced HNSCC undergoing chemo-radiotherapy in which 53 out of 244 (21.7%) had grade 3 iENE [21]. This is an important consideration, because gross invasion of adjacent structures is the single most accurate radiological predictor of pENE in HNSCC [28, 29] and this iENE subgroup was rarely encountered in the PATHOS trial. Including patients with clinically overt ENE is likely to have improved the overall accuracy of the overall correlation between iENE and pENE. However, we will never have that data as those patients rarely undergo surgery and pENE assessment. One could argue that iENE assessment is most useful when ENE is not clinically apparent and our assessment of its accuracy best reflects that scenario.

As participants were recruited from different UK sites over a 9-year period, there was some variation in the quality of cross-sectional imaging (CT and/or MRI) available for review. Over one in six examinations (18.6%) were deemed suboptimal for iENE assessment, mirroring the ‘real world’ situation where imaging for cancer staging is performed in multiple locations by different healthcare providers. A recent review summarises the wide range in diagnostic performance of iENE on CT and/or MRI compared with pENE in patients with HNSCC, which is attributed to shortcomings in radiological assessment (including imaging protocols, differing criteria for iENE, and inter-observer variability), as well as pathological assessment [30]. This highlights the need to address comparative assessment of iENE and pENE in the context of prospective clinical trials (such as PATHOS). A final consideration is that our cohort is not complete (12 slide sets could not be found and pENE consensus could not be reached for one case) and, of the remaining 66 cases that were pENE+ on local pathology reports, only 55 were judged to be pENE+ by central review, so our estimates for sensitivity have wider confidence intervals than anticipated.

## Conclusion

Taken together, these data demonstrate that iENE has good specificity but poor sensitivity for predicting pENE in patients with HPV-positive OPSCC undergoing transoral surgery in PATHOS, and suggest that iENE alone should not be used to rule out a primary surgery treatment approach. 26.0% of cases with no pENE documented on their post-operative pathology CRFs had iENE identified on imaging (iENE + /pENE- cases i.e. false positives), suggesting that other histological features may mimic ENE on imaging. Inter-observer variability exists in scoring iENE which can be substantially reduced by sharing experiences, and using agreed, clearly defined parameters for scoring iENE. Challenges for iENE assessment include suboptimal imaging and iatrogenic changes from recent biopsy; optimising imaging protocols, collecting prospective data on the role of US, and taking biopsy-changes into account are important future considerations. The data and exemplars provided in this manuscript will help to inform how ENE assessment is incorporated into future TNM staging protocols for HPV-positive OPCC and adopted into clinical practice across the world.

## Supplementary information

Supplementary material

## Data Availability

The Centre for Trials Research is a signatory of AllTrials and aims to make its research data available wherever possible. Data and sample requests undergo a Centre for Trials Research review process to ensure that the proposal complies with patient confidentiality, regulatory and ethical approvals and any terms and conditions associated with the data and/or samples: https://www.cardiff.ac.uk/centre-for-trials-research/collaborate-with-us/data-requests.

## Code availability

The STATA code is available from the corresponding author upon request.
