# Supplementary material for: Correlation between imaging-detected and pathological extranodal extension in a randomised trial in Human Papillomavirus-positive oropharyngeal cancer
Source: Br J Cancer. 2025 Nov 27;134(3):428–38. doi: 10.1038/s41416-025-03291-z (PMC12852850; doi:10.1038/s41416-025-03291-z)
Supplement: Supplementary file 1 — Supplementary material [file 41416_2025_3291_MOESM1_ESM.docx]

*Supplementary Figure S1: PATHOS Schema*


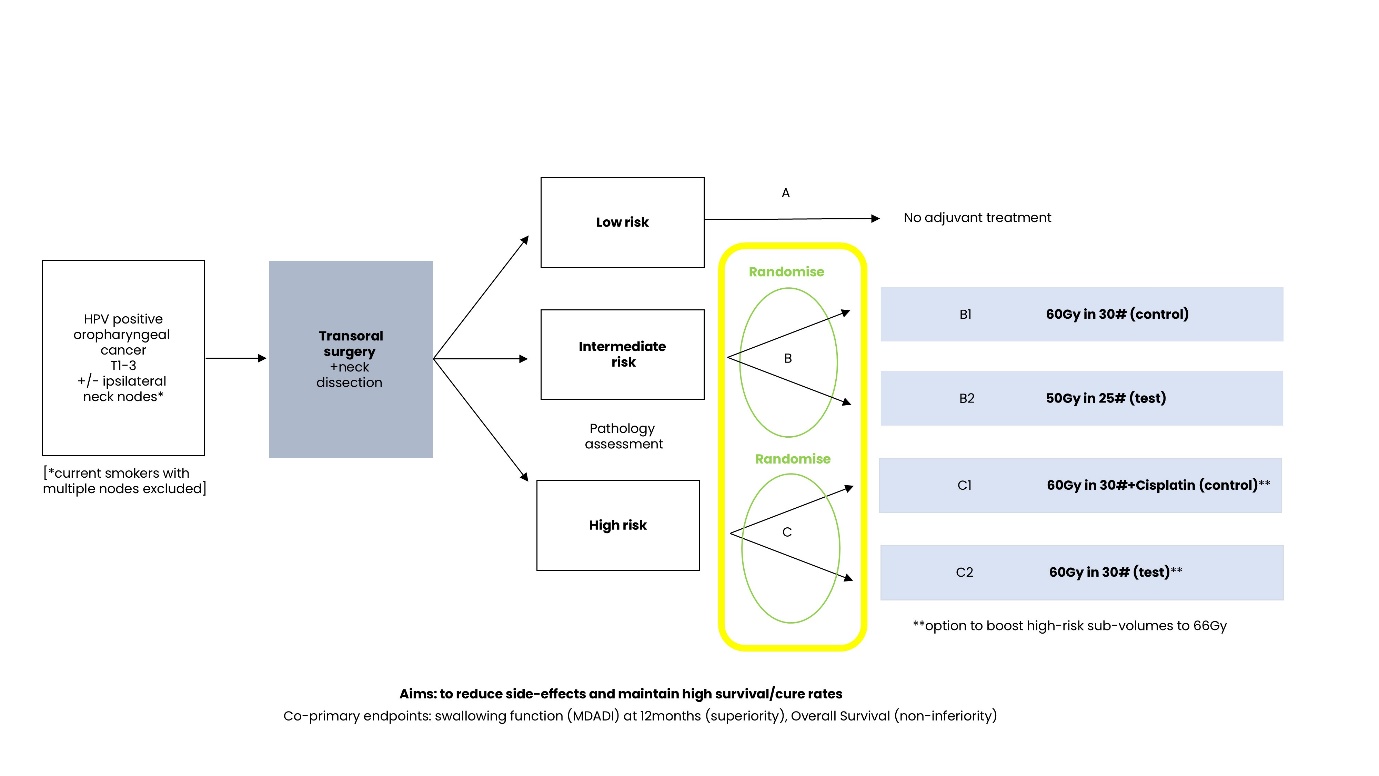


Inclusion: biopsy proven OPSCC, T1-T3 primaries, ipsilateral cervical or no nodal metastases, suitable for transoral resection and neck dissection.

Exclusion: T4 primaries, bilateral neck disease, nodes >6cm, and/or distant metastatic disease.

Risk-group allocation and randomisation: post-operatively, patients are allocated into one of 3 risk groups based on the presence or absence of histological risk factors for recurrence:

- **Group A**: no risk factors, no adjuvant treatment, as per standard of care
- **Group B**: intermediate risk factors (metastasis in a single or multiple ipsilateral nodes >3 cm and ≤6 cm, pT3 category or any pT category with a close [1 to ≤5 mm] margin, perineural and/or vascular invasion). Randomised 1:1 to receive adjuvant radiotherapy over 6 weeks (60 Gray [Gy] in 30 fractions: control arm B1) or 5 weeks (50Gy in 25 fractions: de-intensified test arm B2)
- **Group C**: high risk factors (pathological ENE (pENE) and/or a positive [<1mm] margin around the primary). Randomised 1:1 to receive adjuvant chemo-radiotherapy (60-66Gy in 30 fractions with Cisplatin chemotherapy: control arm C1) or radiotherapy alone (60-66Gy in 30 fractions: de-intensified test arm C2).

*Supplementary Table S1. Inter rater agreement of iENE – Wales/Liverpool team*

| **First reading** | | | | | | | | |
| --- | --- | --- | --- | --- | --- | --- | --- | --- |
| **Cases** | | | | | | **Agreement measures** | | |
|  | **Reader 2 – iENE score** | | | | |  |  |  |
|  | **0** | **1** | **2** | **3** | **Total** |  | **Agreement (95%CI)** | **Gwet’s AC (95%CI)** |
| **Reader 1 – iENE score** |  |  |  |  |  |  |  |  |
| **0** | 39 | 49 | 35 | 1 | **124** | 4 categories | 0.48 (0.41-0.54) | 0.32 (0.23-0.41) |
| **1** | 0 | 16 | 5 | 3 | **24** | Binary: 0 vs 1/2/3 | 0.60 (0.53-0.67) | 0.24 (0.10-0.38) |
| **2** | 0 | 10 | 43 | 8 | **61** | Binary: 0/1 vs 2/3 | 0.75 (0.69-0.80) | 0.52 (0.40-0.64) |
| **3** | 0 | 0 | 0 | 3 | **3** |  |  |  |
| **Total** | **39** | **75** | **83** | **15** | **212** |  |  |  |
| **Second reading** | | | | | | | | |
| **Cases** | | | | | | **Agreement measures** | | |
|  | **Reader 2 – iENE score** | | | | |  |  |  |
|  | **0** | **1** | **2** | **3** | **Total** |  | **Agreement (95%CI)** | **Gwet’s AC (95%CI)** |
| **Reader 1 – iENE score** |  |  |  |  |  |  |  |  |
| **0** | 127 | 4 | 8 | 0 | 139 | 4 categories | 0.93 (0.90-0.97) | 0.92 (0.88-0.96) |
| **1** | 0 | 17 | 0 | 0 | 17 | Binary: 0 vs 1/2/3 | 0.93 (0.80-0.94) | 0.88 (0.81-0.94) |
| **2** | 2 | 0 | 51 | 0 | 53 | Binary: 0/1 vs 2/3 | 0.95 (0.92-0.98) | 0.92 (0.87-0.97) |
| **3** | 0 | 0 | 0 | 3 | 3 |  |  |  |
| **Total** | 129 | 21 | 59 | 3 | 212 |  |  |  |

*Supplementary Table S2. Inter rater agreement of iENE – Newcastle team*

| **First reading** | | | | | | | | |
| --- | --- | --- | --- | --- | --- | --- | --- | --- |
| **Cases** | | | | | | **Agreement measures** | | |
|  | **Reader 2 – iENE score** | | | | |  |  |  |
|  | **0** | **1** | **2** | **3** | **Total** |  | **Agreement (95%CI)** | **Gwet’s AC (95%CI)** |
| **Reader 1 – iENE score** |  |  |  |  |  |  |  |  |
| **0** | 26 | 12 | 4 | 0 | 42 | **4 categories** | 0.52 (0.41-0.63) | 0.38 (0.23-0.53) |
| **1** | 3 | 6 | 2 | 0 | 11 | **Binary: 0 vs 1/2/3** | 0.72 (0.62-0.82) | 0.45 (0.24-0.65) |
| **2** | 1 | 4 | 9 | 2 | 16 | **Binary: 0/1 vs 2/3** | 0.78 (0.69-0.88) | 0.63 (0.45-0.81) |
| **3** | 2 | 4 | 4 | 0 | 10 |  |  |  |
| **Total** | **32** | **26** | **19** | **2** | **79** |  |  |  |
| **Second reading** | | | | | | | | |
| **Cases** | | | | | | **Agreement measures** | | |
|  | **Reader 2 – iENE score** | | | | |  |  |  |
|  | **0** | **1** | **2** | **3** | **Total** |  | **Agreement (95%CI)** | **Gwet’s AC (95%CI)** |
| **Reader 1 – iENE score** |  |  |  |  |  |  |  |  |
| **0** | 46 | 1 | 2 | 0 | 49 | **4 categories** | 0.84 (0.75-0.92) | 0.80 (0.70-0.90) |
| **1** | 5 | 9 | 1 | 0 | 15 | **Binary: 0 vs 1/2/3** | 0.89 (0.81-0.96) | 0.79 (0.65-0.93) |
| **2** | 1 | 1 | 11 | 1 | 14 | **Binary: 0/1 vs 2/3** | 0.92 (0.86-0.98) | 0.89 (0.80-0.98) |
| **3** | 0 | 1 | 0 | 0 | 1 |  |  |  |
| **Total** | 52 | 12 | 14 | 1 | 79 |  |  |  |

*Supplementary Table S3. Inter rater agreement of iENE for cases with MRI available*

| **Second reading** | | | | | | | | |
| --- | --- | --- | --- | --- | --- | --- | --- | --- |
| **Cases** | | | | | | **Agreement measures** | | |
|  | **Reader 2 – iENE score** | | | | |  |  |  |
|  | **0** | **1** | **2** | **3** | **Total** |  | **Agreement (95%CI)** | **Gwet’s AC (95%CI)** |
| **Reader 1 – iENE score** |  |  |  |  |  |  |  |  |
| **0** | 160 | 5 | 8 | 0 | **173** | 4 categories | 0.93 (0.90-0.96) | 0.91 (0.87-0.95) |
| **1** | 1 | 22 | 1 | 0 | **24** | Binary: 0 vs 1/2/3 | 0.93 (0.90-0.96) | 0.88 (0.82-0.94) |
| **2** | 3 | 0 | 56 | 0 | **59** | Binary: 0/1 vs 2/3 | 0.95 (0.92-0.98) | 0.92 (0.88-0.96) |
| **3** | **0** | **1** | **0** | **3** | **4** |  |  |  |
| **Total** | **181** | **28** | **65** | **3** | **260** |  |  |  |

*Supplementary Table S4. Inter rater agreement of pENE as 3 level categorical*

|  | **Reader 2** | | | |
| --- | --- | --- | --- | --- |
|  | **None** | **Minor** | **Major** | **Total** |
| **Reader 1** |  |  |  |  |
| **None** | 0 | 0 | 0 | **0** |
| **Minor** | 0 | 6 | 1 | **7** |
| **Major** | 4 | 6 | 27 | **37** |
| **Total** | **4** | **12** | **28** | **44** |

*Supplementary Table S5. Impact on sensitivity and specificity of iENE (for pENE>0) of excluding suboptimal imaging and recent core biopsy*

|  |  |  | **All patients** | **Excluding suboptimal imaging** | **Excluding core biopsy <10 days ago** | **Excluding core biopsy <30 days ago** | **Excluding core biopsy <50 days ago** | **Excluding core biopsy <30 days ago and suboptimal imaging** |
| --- | --- | --- | --- | --- | --- | --- | --- | --- |
|  |  | **n*** | 55 | 47 | 42 | 34 | 31 | 30 |
|  |  | **N**** | 278 | 227 | 214 | 181 | 172 | 115 |
| **iENE** | **0 or 1/2/3** | Agreement  Gwet’s AC | 56.4 (42.3-69.7)  74.0 (67.7-79.6) | 55.3 (40.1-69.8)  77.8 (71.0-83.6) | 54.8 (38.7-70.2)  71.5 (64.1-78.1) | 58.8 (40.7-75.4)  74.1 (66.2-81.0) | 58.6 (39.1-75.4)  75.2 (67.2-82.1) | 59.4 (40.6-76.3)  79.1 (70.6-86.1) |
|  | **0/1 or 2/3** | Agreement  Gwet’s AC | 43.6 (30.3-57.7)  82.5 (76.9-87.2) | 40.4 (26.4-55.7)  85.6 (79.6-90.3) | 45.2 (29.8-61.3)  80.8 (74.1-86.4) | 52.9 (35.1-70.2)  83.0 (76.0-88.7) | 51.6 (33.1-69.8)  83.0 (75.7-88.8) | 50.0 (31.3-68.7)  87.8 (80.4-93.2) |

*number of patients with pENE>0

**number of patients after any exclusions

*Supplementary Table S6. Sub-optimal imaging and core biopsy prior to imaging in iENE/pENE discordant and concordant cases*

|  | **Discordant cases** | | **Concordant cases** |
| --- | --- | --- | --- |
|  | **iENE-/pENE+** | **iENE+/pENE-** | **iENE+/pENE+ or iENE-/pENE-** |
| **N** | 24 | 58 | 196 |
| **Sub-optimal imaging** | 3 (12.5%) | 17 (29.3%) | 30 (15.3%) |
| **Core biopsy <30 days prior to imaging** | 10 (41.7%) | 20 (34.5%) | 67 (34.2%) |
